# The First Report of Genetic and Structural Diversities in the *SPRN* Gene in the Horse, an Animal Resistant to Prion Disease

**DOI:** 10.3390/genes11010039

**Published:** 2019-12-28

**Authors:** Sae-Young Won, Yong-Chan Kim, Seon-Kwan Kim, Byung-Hoon Jeong

**Affiliations:** 1 Korea Zoonosis Research Institute, Jeonbuk National University, Iksan, Jeonbuk 54531, Korea; gkfh32@jbnu.ac.kr (S.-Y.W.); kych@jbnu.ac.kr (Y.-C.K.); lonicera@jbnu.ac.kr (S.-K.K.); 2 Department of Bioactive Material Sciences and Institute for Molecular Biology and Genetics, Jeonbuk National University, Jeonju, Jeonbuk 54896, Korea

**Keywords:** prion, shadow of prion protein gene (*SPRN*), polymorphisms, horse, Sho

## Abstract

Prion diseases are fatal neurodegenerative diseases and are characterized by the accumulation of abnormal prion protein (PrP^Sc^) in the brain. During the outbreak of the bovine spongiform encephalopathy (BSE) epidemic in the United Kingdom, prion diseases in several species were reported; however, horse prion disease has not been reported thus far. In previous studies, the shadow of prion protein (Sho) has contributed to an acceleration of conversion from normal prion protein (PrP^C^) to PrP^Sc^, and the shadow of prion protein gene (*SPRN*) polymorphisms have been significantly associated with the susceptibility of prion diseases. We investigated the genotype, allele and haplotype frequencies of the *SPRN* gene using direct sequencing. In addition, we analyzed linkage disequilibrium (LD) and haplotypes among polymorphisms. We also investigated LD between *PRNP* and *SPRN* single nucleotide polymorphisms (SNPs). We compared the amino acid sequences of Sho protein between the horse and several prion disease-susceptible species using ClustalW2. To perform Sho protein modeling, we utilized SWISS-MODEL and Swiss-PdbViewer programs. We found a total of four polymorphisms in the equine *SPRN* gene; however, we did not observe an in/del polymorphism, which is correlated with the susceptibility of prion disease in prion disease-susceptible animals. The *SPRN* SNPs showed weak LD value with *PRNP* SNP. In addition, we found 12 horse-specific amino acids of Sho protein that can induce significantly distributional differences in the secondary structure and hydrogen bonds between the horse and several prion disease-susceptible species. To the best of our knowledge, this is the first report regarding the genetic and structural characteristics of the equine *SPRN* gene.

## 1. Introduction

Prion diseases are fatal neurodegenerative diseases that cause death due to brain accumulation of abnormal prion protein (PrP^Sc^), which is characterized by proteinase K resistance [1,2]. Bovine spongiform encephalopathy (BSE) in cattle was first reported in the United Kingdom in 1986 and spread throughout the cattle industry in the United Kingdom through contaminated feedstuff [3,4]. According to statistics from the World Organisation for Animal Health (http://www.oie.int/en/animal-health-in-the-world/bse-specific-data/), a total of 184,627 cases of BSE were reported in the United Kingdom from 1986 to 2016. During the spread of BSE in the United Kingdom, prion diseases were reported in several species, such as feline spongiform encephalopathy (FSE) in cheetahs, pumas, and cats; transmissible mink encephalopathy (TME) in minks; and chronic wasting disease (CWD) in elk and deer [5]. However, prion diseases in horses have not been reported [6].

Previous studies have reported that the stability of prion protein (PrP) and species-specific amino acids of PrP are related to prion disease susceptibility. The horse has a distinctive amino acid, S167, that contributes to the stability of horse PrP and non-toxicity in the transgenic Drosophila model [7]. In addition, the horse has four important salt bridges and a *β*2-*α*2 loop that contribute to the structural stability of horse PrP. The sequence differences in the loop from horse affect a conformational equilibrium between an exposed and a more buried *β*2-*α*2 loop and this region has been implicated in species barriers to transmission of prion diseases [8,9]. However, spontaneous PrP aggregation was observed in the transgenic mouse model expressing mouse PrP with a horse-specific amino acid substitution of D167S [10]. This result indicates that the protective effect of horse specific amino acid, 167S, is elusive and suggests there is another factor related to the resistance of prion disease in horse beside the properties of the PrP sequence encoded in the prion protein gene (*PRNP*). In a previous study, it was suggested that weak linkage disequilibrium (LD) values between the *PRNP* and *prion-like* protein gene (*PRND*) might be a feature of prion disease-resistant species [11]. In our previous studies, we investigated polymorphisms of the *PRNP* gene and *PRND* gene in the horse, which is known to be a prion disease-resistant animal. Interestingly, only one single nucleotide polymorphism (SNP) in the *PRNP* gene and no SNPs in the *PRND* gene were found in Thoroughbred horses [12,13]. Although the *PRNP* and *PRND* genes have been studied in prion disease-resistant species, a member of the prion gene family called the shadow of prion protein gene (*SPRN*) has not been reported thus far in horses.

The *SPRN* gene encodes the shadow of prion protein (Sho) and has an N-glycosylation site and a glycosylphosphatidylinositol (GPI) anchor like PrP. The hydrophobic domain of the *SPRN* gene is very similar to that of the *PRNP* gene. However, PrP contains a long N-terminal region containing a hydrophobic domain followed by the folded C-terminal domain, whereas Sho has a very similar hydrophobic domain but no folded C-terminal domain. The Sho protein is mainly expressed in the brain and contributes to the conversion of PrP^C^ to PrP^Sc^ [14,15]. In a previous study, in/del (AAAG) polymorphisms in the hydrophobic region of bovine Sho protein were found in atypical BSE-infected cattle [16]. In goats, 602_606insCTCCC of the 3′ untranslated region (UTR) was significantly different between healthy and scrapie-infected cases [17]. In addition, in humans, codon 46 insertion G was found in two of 107 variant Creutzfeldt-Jakob disease (vCJD) patients, whereas the insertion was not detected in 861 controls [18]. This evidence suggests that Sho protein may affect the susceptibility to prion disease.

In this study, we investigated the polymorphisms of the equine *SPRN* gene and analyzed LD and haplotypes among polymorphisms. We also investigated LD between *SPRN* and *PRNP* SNPs. In addition, we compared Sho amino acid sequences of the horse with those of prion disease-susceptible species. Finally, we performed structural modeling of equine Sho protein.

## 2. Materials and Methods

### 2.1. Ethical Statement

The blood samples of 194 different Thoroughbred horses were provided by the Seoul Race Park in the Republic of Korea. All experimental procedures were performed by the Jeonbuk National University Institution Animal Care and Use Committee (CBNU 2016-65).

### 2.2. Genetic Analysis

Genomic DNA was purified from 200 µL of blood by a Blood Genomic DNA Isolation Kit (Qiagen, Valencia, CA, USA). Polymerase chain reaction (PCR) was performed using equine *SPRN* gene-specific primers to identify polymorphisms in the open reading frame (ORF) of exon 2. Amplified PCR product encompassed the region from −137 to +833 of ORF. The sequences of the equine *SPRN* primers were as follows: forward primer 5′-AAT GCT AAG CTT CTG TCC CCG-3′ and reverse primer 5′-CTG GTC TTG GCA CCT CTC TT-3′. A total volume of 25 µL of the PCR mixture included 1 µL of genomic DNA, 10 pmol of each primer, 2.5 µL of 10× *Taq* DNA polymerase buffer, 0.5 µL of a 0.2 µM dNTP mixture, 2.5 µL of 5X band helper and 0.2 µL of *Taq* DNA polymerase (Promega, USA). The PCR conditions were as follows: denaturing at 95 °C for 2 min, 33 cycles of 95 °C for 20 s, 61 °C for 40 s, 72 °C for 1 min 30 s and 1 cycle of 72 °C for 5 min. The PCR products were obtained by the FavorPrep GEL/PCR Purification Mini Kit (FAVORGEN, Taiwan) and directly sequenced with an ABI 3730 sequencer (ABI, Foster City, CA, USA). Sequencing results were visualized using Finch TV software (Geospiza Inc., Seattle, WA, USA).

### 2.3. Statistical Analysis

LD analysis calculated by Lewontin’s D’ (|D’|) and pairwise LD (r^2^) were performed using Haploview version 4.2 (Broad Institute, Cambridge, MA, USA). The Hardy-Weinberg Equilibrium (HWE) test and haplotype analysis were carried out using Haploview version 4.2. The HWE test was calculated by the chi-square test. *p* < 0.05 indicate a significant deviation from HWE. The haplotype analysis was performed with genotyping data of four *SPRN* polymorphisms in 194 Thoroughbred horses. After inputting the genotype information of each animal, the haplotype distribution of the horse *SPRN* gene was predicted by Haploview version 4.2.

### 2.4. Sequence Alignment of Sho Protein among Several Species

The multiple sequence alignment of Sho protein sequences was performed using ClustalW2 (http://www.ebi.ac.uk/Tools/msa/clustalo/) with the protein sequences of human (*Homo sapiens*, NP_001012526.2), cattle (*Bos taurus*, AAY83885.1), sheep (*Ovis aries*, NP_001156033.1), goat (*Capra hircus*, AGU17009.1), red deer (*Cervus elaphus*, ACF24724.1), dog (*Canis lupus familiaris,* XP_022267623.1), and horse (*Equus caballus*, XP_023492126.1). All protein sequences were obtained from GenBank at the National Center for Biotechnology Information (NCBI).

### 2.5. Structural Modeling and Comparison of Sho Protein

Models were built using the SWISS-MODEL program (https://swissmodel.expasy.org/). This modeling was generated using the SWISS-MODEL homology-modeling pipeline. The transcript with the reference sequences was used for homology-based modeling. The templates for homology-based modeling were 2mv9.1 (human, cattle, sheep, goat, and red deer) and 6cgv.2 (dog and horse). The modeling was refined according to the global model quality estimation (GMQE) score and selected high GMQE score model. After modeling, the Swiss-PdbViewer program (https://spdbv.vital-it.ch/) was utilized to analyze the hydrogen bonds and secondary structure of Sho protein.

## 3. Results

### 3.1. Investigation of Polymorphisms of the SPRN Gene in 194 Thoroughbred Horses

**Figure 1:**
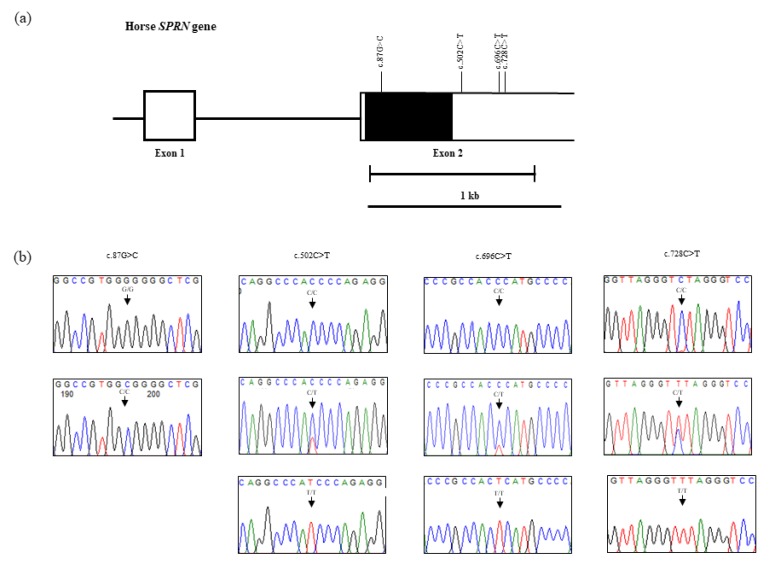
Gene map and polymorphisms of the equine shadow of prion protein gene (*SPRN*) in chromosome 1. (**a**) The open reading frame (ORF) in exon 2 is marked with a black block, and the white blocks represent the 5′ and 3′ untranslated regions (UTRs). The edged horizontal bar indicates the sequenced regions. The four novel polymorphisms found in this study are indicated with vertical lines above the gene. (**b**) Electropherograms of the four novel polymorphisms that were found: c.87G>C, c.502C>T, c.696C>T, and c.728C>T.

We investigated polymorphisms in exon 2 of the *SPRN* gene in Thoroughbred horses using an ABI 3730 automatic direct sequencer. The DNA sequence of the *SPRN* gene in Thoroughbred horses is identical to that of horse registered in GenBank (Gene ID: 111772531). We discovered a total of four novel SNPs: c.87G>C in the ORF and c.502C>T, c.696C>T, and c.728C>T in the 3′ UTR of the *SPRN* gene. All SNPs were novel SNPs and c.87G>C (L39L) was a synonymous SNP (Figure 1a,b). Detailed values of the genotype and allele frequencies of the equine *SPRN* SNPs are described in Table 1. Except for c.87G>C and c.728C>T, c.502C>T and c.696C>T were in HWE (*p* > 0.05). In addition, we measured LD among the four *SPRN* SNPs by |D’| and r^2^ values. The detailed values of LD are described in Table 2. c.87G>C (L39L) showed strong LDs with c.502C>T, c.696C>T, and c.728C>T (Table 2). Furthermore, we performed a haplotype analysis of the four equine *SPRN* SNPs. Six major haplotypes were predicted: GCCC, GCCT, GTTC, CTTT, GTTT, and GCTC. The GCCC haplotype showed the highest (64.7%) frequency in Thoroughbred horses (Table 3).

### 3.2. Investigation of LD between PRNP and SPRN SNPs in 194 Thoroughbred Horses

We also investigated LD between *PRNP* and *SPRN* SNPs using |D**’**| and r^2^ values. The information of *PRNP* SNP was provided from the previous study [13]. The LD analysis was performed with the data of *PRNP* and *SPRN* SNPs obtained from blood samples of 194 different Thoroughbred horses. The detailed values of LD are described in Table 4. Notably, *PRNP* c.525G>C SNP showed weak LD with all *SPRN* SNPs.

### 3.3. The Sequence Alignment of Sho Protein among Several Species

**Figure 2:**
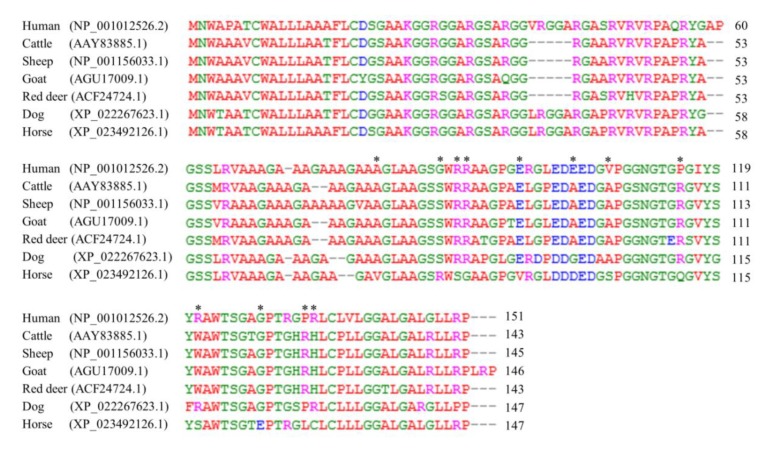
Comparisons of amino acid sequences of the Sho protein among several species. Comparison of amino acid sequences of Sho protein in human (NP_001012526.2), cattle (AAY83885.1), sheep (NP_001156033.1), goat (AGU17009.1), red deer (ACG274724.1) and horse (XP_023492126.1). Amino acids were aligned by the ClustalW2 program. Colors indicate the chemical properties of amino acids as follows: blue: acidic, red: nonpolar aliphatic, magenta: basic, and green: hydroxyl, sulfhydryl, amine, and glycine. Asterisks indicate differences in amino acids between the horse and other species.

We carried out multiple sequence alignment to find differences in the equine Sho protein sequence compared to that of other animals, which are susceptible to TSEs (human, cattle, sheep, goat, and red deer) and resistant to TSEs (dog). We found a total of 12 horse-specific amino acids: valine (V) in codon 77, arginine (R) in codon 84, serine (S) in codon 86, glycine (G) in codon 87, valine (V) in codon 93, aspartic acid (D) in codon 99, serine (S) in codon 103, glutamine (Q) in codon 111, serine (S) in codon 117, glutamic acid (E) in codon 124, leucine (L) in codon 129, and cysteine (C) in codon 130 (Figure 2).

### 3.4. Structural Modeling of Sho Protein and Comparisons with Several Species

**Figure 3:**
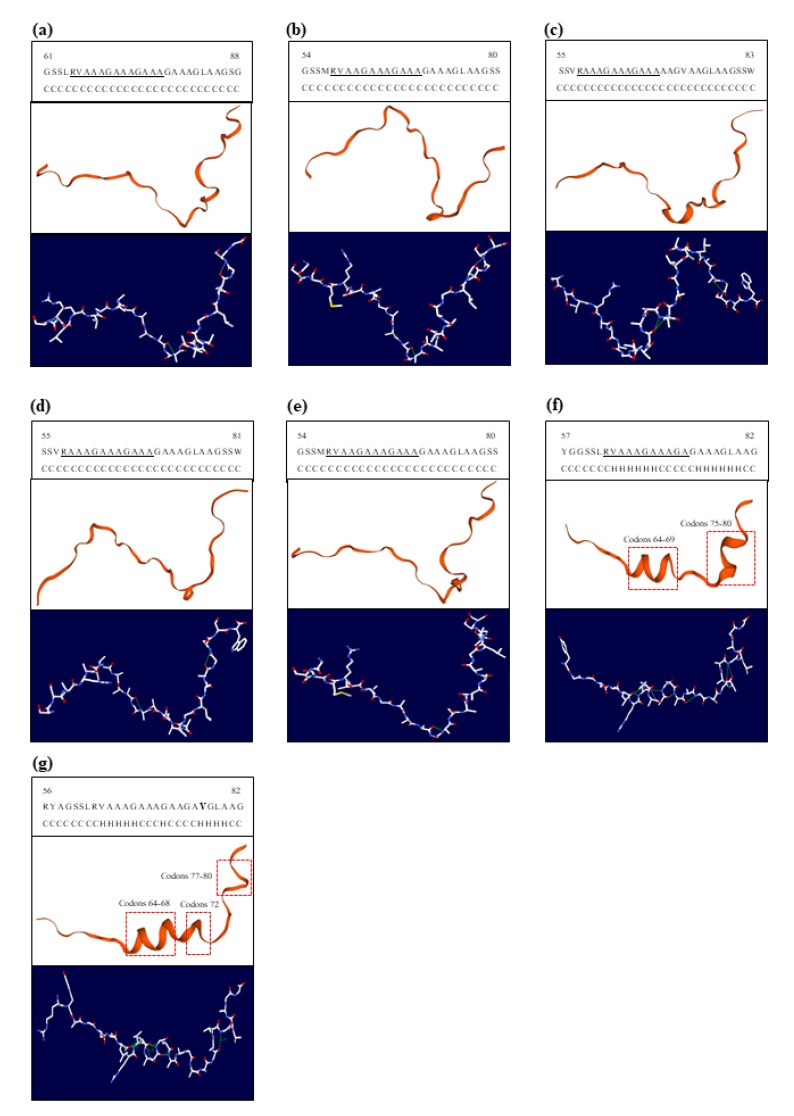
Homology-based modeling of Sho protein among several species. Upper panels indicate a secondary structure predicted by the Swiss-PdbViewer. Middle panels indicate three-dimensional structures of Sho protein analyzed by the SWISS-MODEL program. Lower panels indicate the ball-and-stick model and hydrogen bonds of the Sho protein analyzed by Swiss-PdbViewer. (**a**), Sho protein of human; (**b**), Sho protein of cattle; (**c**), Sho protein of sheep; (**d**), Sho protein of goat; (**e**), Sho protein of red deer; (**f**), Sho protein of dog; (**g**), Sho protein of horse. The red dotted lines indicate α-helices. Green dotted lines indicate hydrogen bonds. Underlines indicate the binding site f PrP. Bold text indicates horse-specific amino acids. C: coil; H: alpha-helix.

We performed modeling and secondary structure prediction of Sho protein to investigate the structural differences between horses and prion disease-susceptible species (Figure 3). Interestingly, we observed an α helix structure in codons 64–68, 72 and 77–80 in the equine Sho protein (Figure 3g). We also observed an α helix structure in codons 64–69 and 75–80 in another prion disease-resistant animal, dog. However, the α helix structure of Sho protein was not observed in the prion disease-susceptible species, including cattle, sheep, goat, and red deer. We also performed an H-bond analysis using the Swiss-PdbViewer program (https://spdbv.vital-it.ch/). There was a significant difference in the distribution and number (horse: 6; dog: 8; human: 2; cattle: 2. sheep; 3, goat: 2, and red deer: 2) of hydrogen bonds (green dotted lines) between prion disease-resistant species (horses and dog) (Figure 3f,g) and prion disease-susceptible species (Figure 3a–e). The detailed information of the H-bonds is described in Table 5.

## 4. Discussion

According to previous studies, horses have been considered a prion disease-resistant species [6]. Several studies in horses have tried to find distinctive features that may be associated with the mechanism of prion disease resistance [9,19]. However, there is not enough research on prion family genes that can affect the susceptibility of prion disease. In the horse, one and no polymorphisms have been reported in the *PRNP* and *PRND* genes, respectively [13]. In previous studies, a strong LD value between *PRNP* and *PRND* gene polymorphisms has been reported in prion disease-susceptible species [20,21,22]. However, the LD value between SNPs of these two genes in horses could not be estimated, as there are no polymorphisms in the equine *PRND* gene [12]. In addition, a low LD value was observed between *PRNP* and *PRND* gene polymorphisms in another prion disease-resistant species: the dog [11]. Since these genetic characteristics in prion disease-resistant species were apparently different from the strong LD observed in prion disease-susceptible species, we investigated another prion protein family gene, the *SPRN* gene, in the horse.

We found a total of four novel SNPs: one synonymous SNP in the ORF and three polymorphisms in the 3′ UTR. Except for rare polymorphisms including c.87G>C and c.728C>T, all polymorphisms were in HWE (Table 1). Notably, nonsynonymous SNPs and in/del polymorphisms have not been identified in the ORF of the equine *SPRN* gene. Since previous studies have reported that in/del polymorphisms of the *SPRN* gene were associated with the susceptibility of prion disease in cattle, sheep, and humans, no in/del polymorphism of the equine *SPRN* gene is noteworthy. In addition, in/del polymorphisms in the 3′ UTR have not been identified in horses, unlike the detection of this polymorphism in goats, a prion disease-susceptible animal. Since polymorphisms of promoter or 3′UTR can modulate the expression level of genes, it needs further investigation [17,23,24]. Furthermore, we investigated LD between *SPRN* and *PRNP* SNPs because the LD value among prion family genes has showed distinct features in prion disease-resistant animals [11]. Interestingly, all *SPRN* SNPs showed a weak LD with *PRNP* SNP. Further research is needed to confirm whether it is characteristic of prion resistant species.

In recent studies, the serine residue at codon 167 of the equine PrP, which is a horse-specific amino acid, contributes to the extraordinary stable structure of equine PrP and the resistance of prion disease [7]. Since a horse-specific amino acid showed distinct features in prion disease-resistant animals, we tried to find horse-specific amino acids in the Sho protein. Using multiple alignment programs with amino acids from several species, we identified a total of 12 horse-specific amino acids (Figure 2). To investigate whether these differences in equine Sho protein induce a structural difference in Sho protein with several species, we analyzed the 3D structure of Sho protein using the SWISS-MODEL program. Notably, Sho protein in horses was predicted to have different protein structures compared to that in prion disease-susceptible species. In brief, there are differences in the secondary structure and hydrogen bonds between equine Sho protein and Sho protein in other species. Notably, the difference of structure was found in PrP binding site (Figure 3). In the previous study, identical peptide sequences can show different conformation in different proteins [25]. Thus, different Sho sequences among species, could have caused different conformation between prion disease-susceptible animals and prion disease-resistant animals. Because structural differences can affect the function of a protein, these results are noteworthy. According to previous studies, Sho protein can accelerate the conversion from PrP^C^ to PrP^Sc^ in prion disease-susceptible species; thus, further studies of the association between the apparent structural differences identified in the present study and the prion disease-related function of Sho protein are needed in the future [15]. The potential effect of horse Sho structure on the ability to bind PrP is needed to be verified using Immunoprecipitation (IP) or co-IP in the future.

## 5. Conclusions

In conclusion, we found a total of four SNPs in the *SPRN* gene: one synonymous SNP and three SNPs in the 3′ UTR. In addition, the *SPRN* SNPs showed weak LD with *PRNP* SNP. Next, we found a total of 12 horse-specific amino acids via protein alignment. Finally, significant structural differences, such as secondary structures and hydrogen bonds, were identified by comparative analysis of the 3D structure of Sho protein among several species. To the best of our knowledge, this was the first study regarding the *SPRN* gene in horses.

## Figures and Tables

**Table 1:** Genotype and allele frequencies of the shadow of the prion protein gene (*SPRN*) polymorphisms in Thoroughbred horses.

	Genotype Frequency, *n* (%)			Allele Frequency, *n* (%)		HWE
c.87G>C	GG	GC	CC	G	C	0
	191 (98.5)	0 (0)	3 (1.5)	382 (98.5)	6 (1.5)	
c.502C>T	CC	CT	TT	C	T	0.0223
	170 (87.6)	21 (10.9)	3 (1.5)	361 (93.1)	27 (6.9)	
c.696C>T	CC	CT	TT	C	T	0.4665
	168 (86.6)	23 (11.9)	3 (1.5)	359 (92.5)	29 (7.5)	
c.728C>T	CC	CT	TT	C	T	0
	108 (55.7)	54 (27.8)	32 (16.5)	270 (69.6)	118 (30.4)	

HWE: Hardy-Weinberg Equilibrium.

**Table 2:** Linkage disequilibrium (LD) among four polymorphisms of the shadow of prion protein gene (*SPRN*) gene in Thoroughbred horses.

	|D’|			
r^2^	c.87G>C	c.502C>T	c.696C>T	c.728C>T
c.87G>C	-	1.0	1.0	1.0
c.502C>T	0.21	-	0.919	0.238
c.696C>T	0.194	0.782	-	0.138
c.728C>T	0.036	0.01	0.004	-

The figures above the diagonal indicate |D**’**| value. The figures below the diagonal indicate r^2^ value.

**Table 3:** Haplotype frequency of four shadow of prion protein gene (*SPRN*) polymorphisms in Thoroughbred horses as predicted by Haploview.

Haplotype	Thoroughbred (*n* = 388)
GCCC	251 (0.647)
GCCT	105 (0.271)
GTTC	14 (0.036)
CTTT	6 (0.016)
GTTT	5 (0.013)
GCTC	4 (0.010)
Others	3 (0.007)

**Table 4:** Linkage disequilibrium (LD) between single nucleotide polymorphisms (SNPs) of *SPRN* and *PRNP* gene with D’ and r^2^ value in Thoroughbred horses.

	|D’|				
r^2^	*PRNP* c.525G>C	*SPRN* c.87G>C	*SPRN* c.502C>T	*SPRN* c.696C>T	*SPRN* c.728C>T
*PRNP* c.525G>C	-	0.102	0.314	0.195	0.037
*SPRN* c.87G>C	0.001	-	1.0	1.0	1.0
*SPRN* c.502C>T	0.022	0.247	-	0.905	0.289
*SPRN* c.696C>T	0.009	0.226	0.748	-	0.167
*SPRN* c.728C>T	0.001	0.042	0.014	0.005	-

The figures above the diagonal indicate |D**’**| value. The figures below the diagonal indicate r^2^ value.

**Table 5:** Detailed information of Sho protein in several species analyzed by SWISS-MODEL and Swiss-PdbViewer programs.

Common Name	Scientific Name	GenBank ID	Location of α-Helix Structure	Location of Hydrogen Bonds	Number of Hydrogen Bonds
Human	*Homo sapiens*	NP_001012526.2	Not found	A73–A75, A85–S87	2
Cattle	*Bos taurus*	AAY83885.1	Not found	A65–A67, A77–S79	2
Sheep	*Ovis aries*	NP_001156033.1	Not found	G66–A69, G66–A70, A79–S81	3
Goat	*Capra hircus*	AGU17009.1	Not found	A65–A67, A77–S79	2
Red deer	*Cervus elaphus*	ACG274724.1	Not found	A65–A67, A77–S79	2
Dog	*Canis lupus familiaris*	XP_022267623.1	Codons 64–69, 75–80	L62–V64, L62–A65, V64–A67, V64–G68, A67–A70, A70–G72, G74–G78, A75–G78	8
Horse	*Equus caballus*	XP_023492126.1	Codons 64–68, 72, 77–80	L62–V64, L62–A65, V64–A67, C64–G68, A67–A70, G75–G78	6
